# Supplementary material for: Effectiveness of an Intervention Programme on Adherence to the Mediterranean Diet in a Preschool Child: A Randomised Controlled Trial
Source: Nutrients. 2022 Apr 7;14(8):1536. doi: 10.3390/nu14081536 (PMC9025428; doi:10.3390/nu14081536)
Supplement: Supplementary file 1 [file nutrients-14-01536-s001.zip › nutrients-1609803-SI.pdf]

Table S1. Homogeneity analysis for parental sociodemographic variables.

		Experimental group (n = 65)	Control group (n = 68)	*p* Value
Mean initial age (SD)		31.02 (4.815)	31.32 (4.333)	0.711
Mean father's age (SD)		32.39 (8.023)	32.55 (7.465)	0.908
Mother's level of education, n (%)	Primary	6 (9.8)	7 (10.3)	0.632
	Secondary	41 (67.2)	45 (68.2)	
	University	11 (18.0)	12 (18.2)	
	None	3 (4.9)	2 (3.0)	
Father's level of education, n (%)	Primary	7 (11.9)	7 (11.1)	0.992
	Secondary	39 (66.1)	42 (66.7)	
	University	13 (22.0)	14 (22.2)	
Mother's socioeconomic level, n (%)	No qualification,	3 (7.5)	3 (7.0)	0.884
	Auxiliary	21 (52.5)	23 (53.5)	
	Technical	10 (25.0)	12 (27.9)	
	University	5 (12.5)	5 (11.6)	
	Not working	1 (2.5)	0 (0.0)	
Father's socioeconomic level, n (%)	No qualifications,	6 (10.3)	8 (17.2)	0.971
	Auxiliary	13 (22.4)	15 (23.8)	
	Technical	33 (56.9)	34 (54.0)	
	University	6 (10.3)	6 (9.5)	
Father's ethnicity, n (%)	Mediterranean	45 (73.8)	49 (72.4)	0.975
	Caucasian	4 (6.6)	5 (7.6)	
	American	2 (3.3)	3 (4.5)	
	Asian	1 (1.6)	1 (1.5)	
	Saharan	3 (4.9)	4 (6.1)	
	Gitano Gypsy	6 (9.8)	4 (6.1)	

SD, Standard deviation.
